# Supplementary material for: Deciphering signaling mechanisms and developmental dynamics in extraembryonic mesoderm specification from hESCs
Source: Nat Commun. 2025 May 21;16:4688. doi: 10.1038/s41467-025-59491-x (PMC12095623; doi:10.1038/s41467-025-59491-x)
Supplement: Supplementary file 1 — Supplementary Information [file 41467_2025_59491_MOESM1_ESM.pdf]

Supplementary Information

Deciphering signaling mechanisms and developmental dynamics in extraembryonic mesoderm specification from hESCs

Baohua Niu[1, 2#], Da Wang[1, 2#], Yingjie Hu[1, 2#], Yundi Wang[1, 2#], Gaohui Shi[1, 2#], Zhongying Chen[3], Lifeng Xiang[4], Chi Zhang[1, 2], Xuesong Wei[1, 2], Ruize Kong[4], Hongzhi Cai[1, 2], Weizhi Ji[1, 2, 5]*, Yu Yin[1, 2, 5]*, Tianqing Li[1, 2, 5]*, Zongyong Ai[1, 2, 5]*

This PDF file includes:

Supplementary Figures 1 to 8

Supplementary Table 1

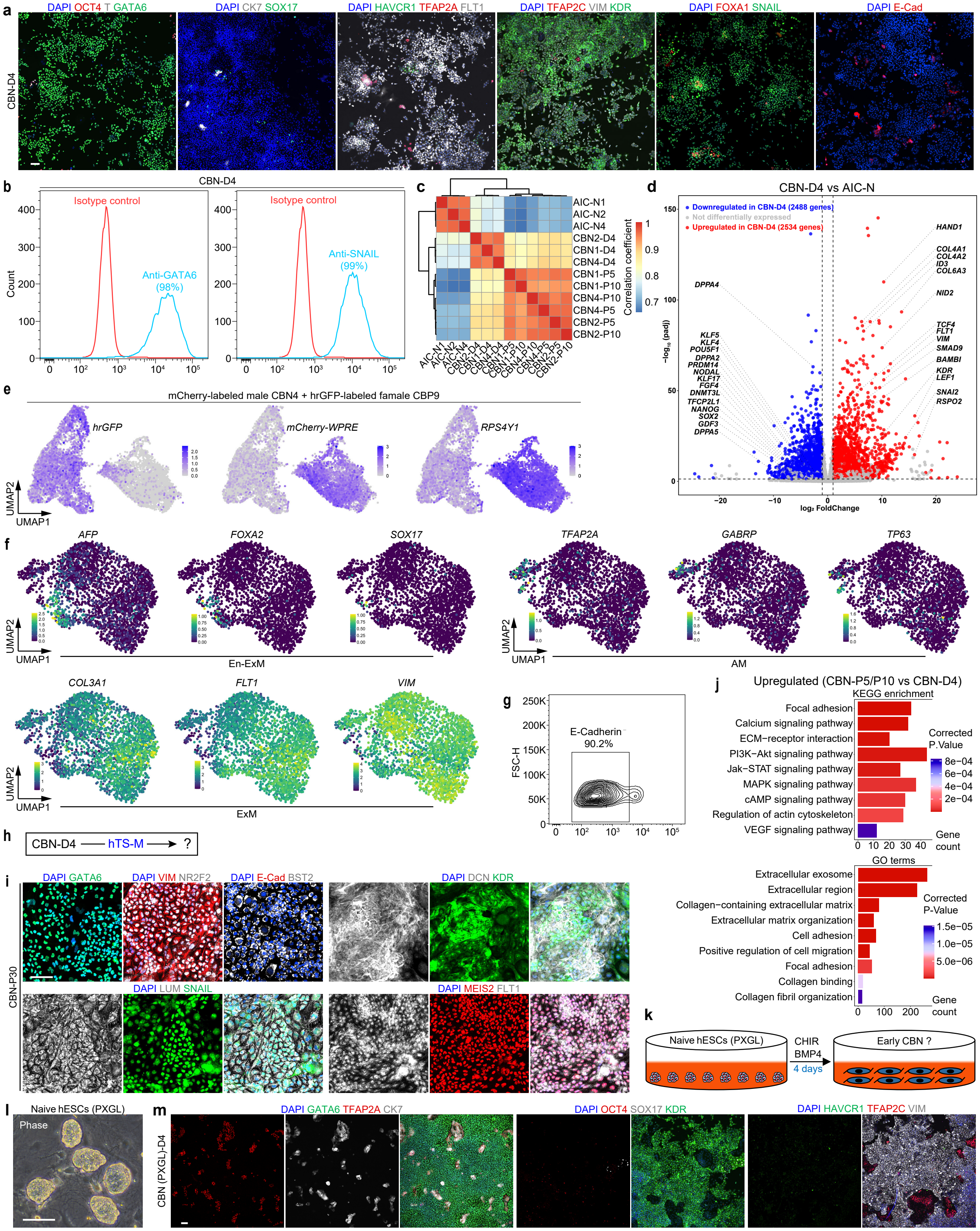
a
DAPI OCT4 T GATA6
DAPI CK7 SOX17
DAPI HAVCR1 TFAP2A FLT1
DAPI TFAP2C VIM KDR
DAPI FOXA1 SNAIL
DAPI E-Cad
CBN-D4
b
CBN-D4
Isotype control
Anti-GATA6 (98%)
Isotype control
Anti-SNAIL (99%)
Count
c
AIC-N1
AIC-N2
AIC-N4
CBN2-D4
CBN1-D4
CBN4-D4
CBN1-P5
CBN1-P10
CBN4-P10
CBN4-P5
CBN2-P5
CBN2-P10
Correlation coefficient
d
CBN-D4 vs AIC-N
Downregulated in CBN-D4 (2488 genes)
Not differentially expressed
Upregulated in CBN-D4 (2534 genes)
HAND1
COL4A1
COL4A2
ID3
COL6A3
NID2
TCF4
FLT1
VIM
SMAD9
BAMBI
KDR
LEF1
SNAI2
RSPO2
DPPA4
KLF5
KLF4
POU5F1
DPPA2
PRDM14
NODAL
KLF17
FGF4
DNMT3L
TFCP2L1
NANOG
SOX2
GDF3
DPPA5
log₂ FoldChange
e
mCherry-labeled male CBN4 + hrGFP-labeled famale CBP9
hrGFP
mCherry-WPRE
RPS4Y1
UMAP1
UMAP2
f
AFP
FOXA2
SOX17
En-ExM
TFAP2A
GABRP
TP63
AM
COL3A1
FLT1
VIM
ExM
g
E-Cadherin⁻
90.2%
FSC-H
h
CBN-D4 —— hTS-M ——→ ?
i
DAPI GATA6
DAPI VIM NR2F2
DAPI E-Cad BST2
DAPI DCN KDR
DAPI LUM SNAIL
DAPI MEIS2 FLT1
CBN-P30
j
Upregulated (CBN-P5/P10 vs CBN-D4)
KEGG enrichment
Focal adhesion
Calcium signaling pathway
ECM−receptor interaction
PI3K−Akt signaling pathway
Jak−STAT signaling pathway
MAPK signaling pathway
cAMP signaling pathway
Regulation of actin cytoskeleton
VEGF signaling pathway
Corrected P.Value
Gene count
GO terms
Extracellular exosome
Extracellular region
Collagen−containing extracellular matrix
Extracellular matrix organization
Cell adhesion
Positive regulation of cell migration
Focal adhesion
Collagen binding
Collagen fibril organization
Corrected P-Value
Gene count
k
Naive hESCs (PXGL)
CHIR
BMP4
4 days
Early CBN ?
l
Naive hESCs (PXGL)
Phase
m
DAPI GATA6 TFAP2A CK7
DAPI OCT4 SOX17 KDR
DAPI HAVCR1 TFAP2C VIM
CBN (PXGL)-D4

Supplementary Fig. 1 | Identification and extended culture of naive hESC-derived ExMs, related to Fig. 1. a Immunofluorescence (IF) staining for various embryonic and extraembryonic lineage markers in CHIR+BMP4-treated naive hESCs on day 4 (CBN-D4). **b** Flow cytometry analysis of GATA6 and SNAIL expression in CBN-D4. **c** Spearman correlation analysis of gene expression patterns in differentiation time courses for CBNs. **d** Volcano plot representing the differentially expressed genes between CBN-D4 and naive (AIC-N) hESCs, $\log_2$ FC ≥ 1 or ≤ -1, padj < 0.05. Two-sided Wald test, *p*-values were adjusted using the Benjamini-Hochberg method. **e** UMAP plots showing expressions of *hrGFP*, *mCherry-WPRE*, and Y-chromosome specific gene *RPS4Y1* according to scRNA-seq data from CB-treated AIC-N4 (CBN4-D4, male, mCherry-labeled) and primed H9 (CBP9-D4, female, hrGFP-labeled) hESCs on day 4. The fluorescent protein genes were knocked into AAVS1 site in hESCs according to the published method[3]. To minimize batch effects and costs in scRNA-seq, we collected the indicated sample pair as pooled sample by mixing equal cell numbers. **f** UMAP plots of the indicated genes expressed in CBN4-D4. ExM, extraembryonic mesoderm; AM, amnion; EnExM, ExM expressing specific endoderm markers. **g** Flow cytometry contour plot of CBN-D4 for E-Cadherin. **h** Schematic of extended culture of D4 CBNs in hTS-M. **i** IF staining for the indicated markers in expandable ExMs at passage 30. **j** Kyoto Encyclopedia of Genes and Genomes (KEGG) and Gene ontology (GO) analysis showing upregulated gene categories in expandable ExMs relative to D4 CBNs. Two-sided hypergeometric test, *p*-values were adjusted using the Benjamini-Hochberg method. **k–m** Schematic diagram (**k**), brightfield (**l**) and IF staining (**m**) images demonstrating naive (PXGL) hESC derivatives induced by CB conditions (unless otherwise specified, naive hESCs were typically cultured in AIC-N medium). All scRNA-seq data in this study are from 10× Genomics platform. Scale bars, 100 μm. Source data are provided as a Source Data file.

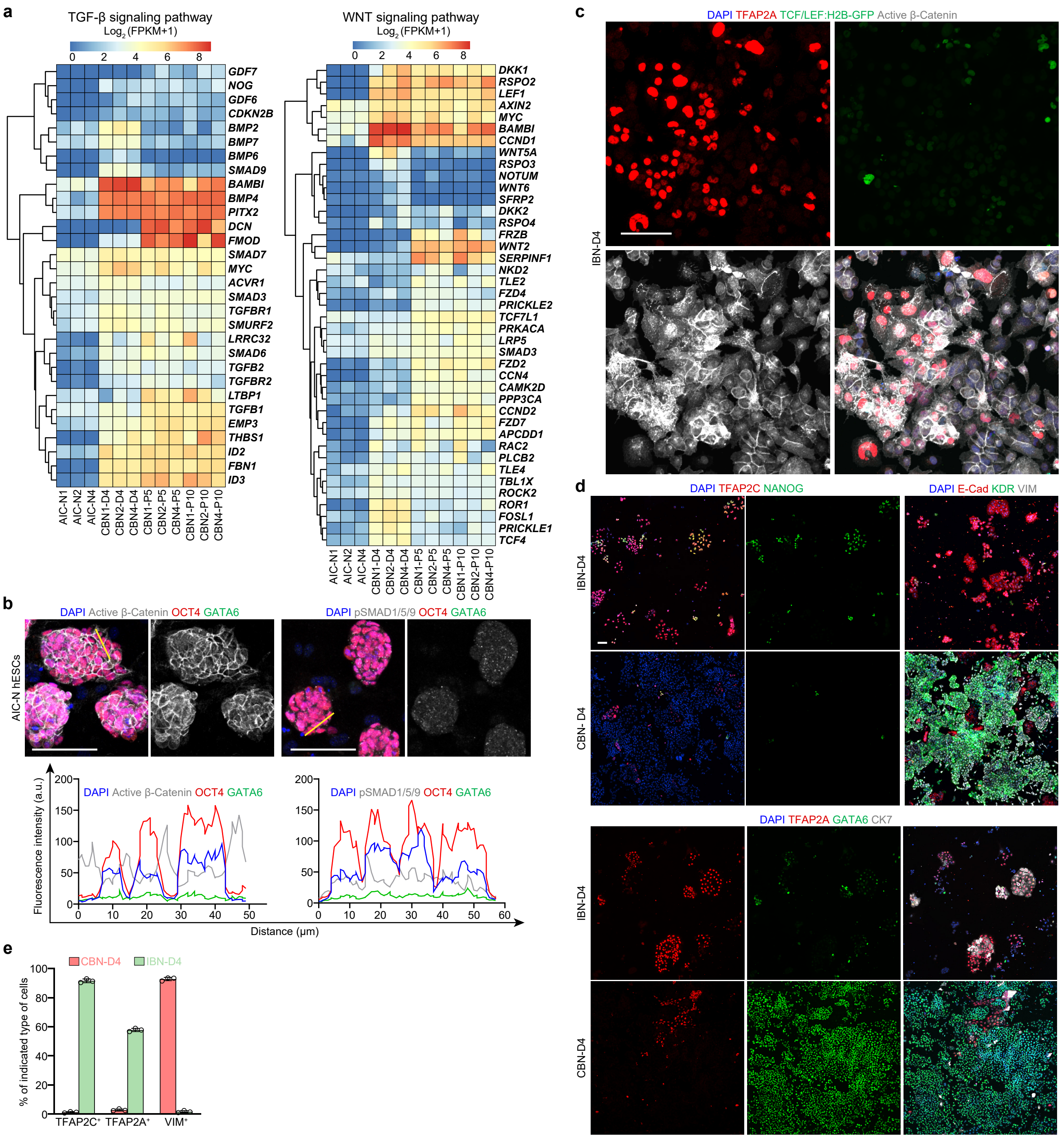
a
TGF-β signaling pathway
Log2 (FPKM+1)
0 2 4 6 8
GDF7
NOG
GDF6
CDKN2B
BMP2
BMP7
BMP6
SMAD9
BAMBI
BMP4
PITX2
DCN
FMOD
SMAD7
MYC
ACVR1
SMAD3
TGFBR1
SMURF2
LRRC32
SMAD6
TGFB2
TGFBR2
LTBP1
TGFB1
EMP3
THBS1
ID2
FBN1
ID3
AIC-N1
AIC-N2
AIC-N4
CBN1-D4
CBN2-D4
CBN4-D4
CBN1-P5
CBN2-P5
CBN4-P5
CBN1-P10
CBN2-P10
CBN4-P10
WNT signaling pathway
Log2 (FPKM+1)
0 2 4 6 8
DKK1
RSPO2
LEF1
AXIN2
MYC
BAMBI
CCND1
WNT5A
RSPO3
NOTUM
WNT6
SFRP2
DKK2
RSPO4
FRZB
WNT2
SERPINF1
NKD2
TLE2
FZD4
PRICKLE2
TCF7L1
PRKACA
LRP5
SMAD3
FZD2
CCN4
CAMK2D
PPP3CA
CCND2
FZD7
APCDD1
RAC2
PLCB2
TLE4
TBL1X
ROCK2
ROR1
FOSL1
PRICKLE1
TCF4
AIC-N1
AIC-N2
AIC-N4
CBN1-D4
CBN2-D4
CBN4-D4
CBN1-P5
CBN2-P5
CBN4-P5
CBN1-P10
CBN2-P10
CBN4-P10
b
DAPI Active β-Catenin OCT4 GATA6
DAPI pSMAD1/5/9 OCT4 GATA6
AIC-N hESCs
Fluorescence intensity (a.u.)
200
150
100
50
0
0 10 20 30 40 50
0 10 20 30 40 50 60
Distance (μm)
c
DAPI TFAP2A TCF/LEF:H2B-GFP Active β-Catenin
IBN-D4
d
DAPI TFAP2C NANOG
DAPI E-Cad KDR VIM
IBN-D4
CBN- D4
DAPI TFAP2A GATA6 CK7
IBN-D4
CBN-D4
e
CBN-D4
IBN-D4
% of indicated type of cells
100
80
60
40
20
0
TFAP2C+
TFAP2A+
VIM+

Supplementary Fig. 2 | WNT signaling governs ExM and AM fate specification in naive hESCs, related to Fig. 2. a Heatmaps showing the expression of genes in TGF-β and WNT signalling pathways in AIC-N hESCs and CBNs. Values represent $\log_2$ (FPKM+1). **b** IF staining (top) and fluorescence intensity profiles (bottom) for signal transducers and lineage markers in AIC-N hESCs. Yellow lines indicate planes used to plot intensity profiles of indicated markers. **c** IF staining of AIC-N2 TCF/LEF:H2B-GFP hESCs grown under IWP2+BMP4 conditions for 4 days with indicated markers. IBN-D4, IWP2+BMP4-treated AIC-N hESCs on D4. **d** IF staining for the indicated markers in IBN-D4 and CBN-D4. **e** Proportion of the indicated cell types in CBN-D4 and IBN-D4. n = 3 independent experiments; data are presented as mean ± SD. Scale bars, 100 μm. Source data are provided as a Source Data file.

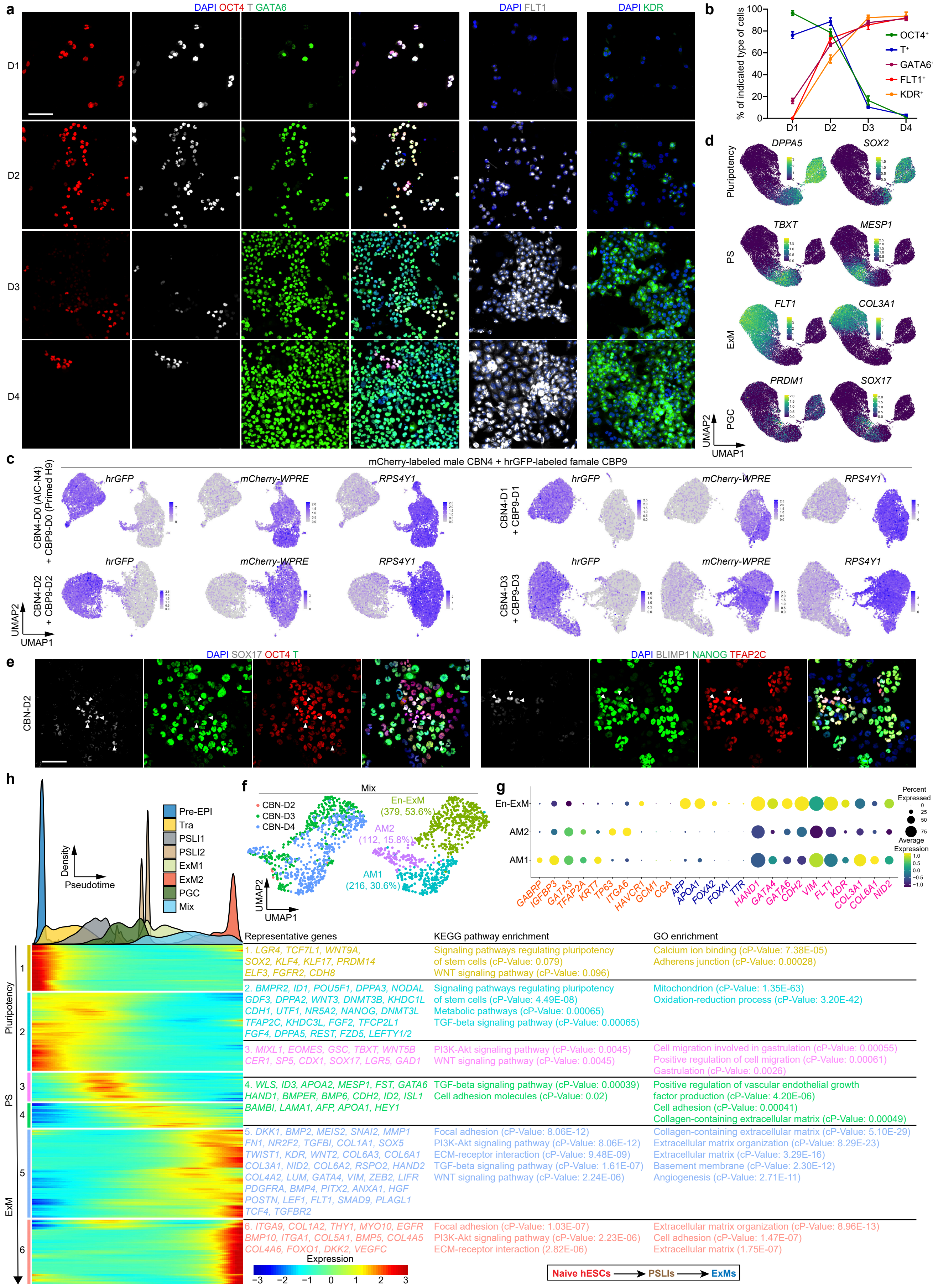

a
DAPI OCT4 T GATA6
DAPI FLT1
DAPI KDR
D1
D2
D3
D4
b
% of indicated type of cells
100
80
60
40
20
0
D1 D2 D3 D4
OCT4+
T+
GATA6+
FLT1+
KDR+
d
Pluripotency
DPPA5
SOX2
PS
TBXT
MESP1
ExM
FLT1
COL3A1
PGC
PRDM1
SOX17
UMAP2
UMAP1
c
mCherry-labeled male CBN4 + hrGFP-labeled famale CBP9
CBN4-D0 (AIC-N4) + CBP9-D0 (Primed H9)
CBN4-D1 + CBP9-D1
CBN4-D2 + CBP9-D2
CBN4-D3 + CBP9-D3
hrGFP
mCherry-WPRE
RPS4Y1
UMAP2
UMAP1
e
DAPI SOX17 OCT4 T
DAPI BLIMP1 NANOG TFAP2C
CBN-D2
h
Pre-EPI
Tra
PSLI1
PSLI2
ExM1
ExM2
PGC
Mix
Density
Pseudotime
f
Mix
CBN-D2
CBN-D3
CBN-D4
En-ExM (379, 53.6%)
AM2 (112, 15.8%)
AM1 (216, 30.6%)
UMAP2
UMAP1
g
En-ExM
AM2
AM1
GABRP IGFBP3 GATA3 TFAP2A KRT7 TP63 ITGA6 HAVCR1 GCM1 CGA AFP APOA1 FOXA2 FOXA1 TTR HAND1 GATA4 GATA6 CDH2 VIM FLT1 KDR COL3A1 COL6A1 NID2
Percent Expressed
0
25
50
75
Average Expression
1.0
0.5
0.0
−0.5
−1.0
Pluripotency
PS
ExM
1
2
3
4
5
6
Representative genes
KEGG pathway enrichment
GO enrichment
1. LGR4, TCF7L1, WNT9A, SOX2, KLF4, KLF17, PRDM14 ELF3, FGFR2, CDH8
Signaling pathways regulating pluripotency of stem cells (cP-Value: 0.079)
WNT signaling pathway (cP-Value: 0.096)
Calcium ion binding (cP-Value: 7.38E-05)
Adherens junction (cP-Value: 0.00028)
2. BMPR2, ID1, POU5F1, DPPA3, NODAL GDF3, DPPA2, WNT3, DNMT3B, KHDC1L CDH1, UTF1, NR5A2, NANOG, DNMT3L TFAP2C, KHDC3L, FGF2, TFCP2L1 FGF4, DPPA5, REST, FZD5, LEFTY1/2
Signaling pathways regulating pluripotency of stem cells (cP-Value: 4.49E-08)
Metabolic pathways (cP-Value: 0.00065)
TGF-beta signaling pathway (cP-Value: 0.00065)
Mitochondrion (cP-Value: 1.35E-63)
Oxidation-reduction process (cP-Value: 3.20E-42)
3. MIXL1, EOMES, GSC, TBXT, WNT5B CER1, SP5, CDX1, SOX17, LGR5, GAD1
PI3K-Akt signaling pathway (cP-Value: 0.0045)
WNT signaling pathway (cP-Value: 0.0045)
Cell migration involved in gastrulation (cP-Value: 0.00055)
Positive regulation of cell migration (cP-Value: 0.00061)
Gastrulation (cP-Value: 0.0026)
4. WLS, ID3, APOA2, MESP1, FST, GATA6 HAND1, BMPER, BMP6, CDH2, ID2, ISL1 BAMBI, LAMA1, AFP, APOA1, HEY1
TGF-beta signaling pathway (cP-Value: 0.00039)
Cell adhesion molecules (cP-Value: 0.02)
Positive regulation of vascular endothelial growth factor production (cP-Value: 4.20E-06)
Cell adhesion (cP-Value: 0.00041)
Collagen-containing extracellular matrix (cP-Value: 0.00049)
5. DKK1, BMP2, MEIS2, SNAI2, MMP1 FN1, NR2F2, TGFBI, COL1A1, SOX5 TWIST1, KDR, WNT2, COL6A3, COL6A1 COL3A1, NID2, COL6A2, RSPO2, HAND2 COL4A2, LUM, GATA4, VIM, ZEB2, LIFR PDGFRA, BMP4, PITX2, ANXA1, HGF POSTN, LEF1, FLT1, SMAD9, PLAGL1 TCF4, TGFBR2
Focal adhesion (cP-Value: 8.06E-12)
PI3K-Akt signaling pathway (cP-Value: 8.06E-12)
ECM-receptor interaction (cP-Value: 9.48E-09)
TGF-beta signaling pathway (cP-Value: 1.61E-07)
WNT signaling pathway (cP-Value: 2.24E-06)
Collagen-containing extracellular matrix (cP-Value: 5.10E-29)
Extracellular matrix organization (cP-Value: 8.29E-23)
Extracellular matrix (cP-Value: 3.29E-16)
Basement membrane (cP-Value: 2.30E-12)
Angiogenesis (cP-Value: 2.71E-11)
6. ITGA9, COL1A2, THY1, MYO10, EGFR BMP10, ITGA1, COL5A1, BMP5, COL4A5 COL4A6, FOXO1, DKK2, VEGFC
Focal adhesion (cP-Value: 1.03E-07)
PI3K-Akt signaling pathway (cP-Value: 2.23E-06)
ECM-receptor interaction (2.82E-06)
Extracellular matrix organization (cP-Value: 8.96E-13)
Cell adhesion (cP-Value: 1.47E-07)
Extracellular matrix (1.75E-07)
Expression
−3 −2 −1 0 1 2 3
Naive hESCs → PSLIs → ExMs

Supplementary Fig. 3 | ExM specification recapitulates the progression from naive pluripotency to PSLI to ExM, related to Fig. 3. a IF staining to demonstrate the dynamic expression of the indicated markers in differentiation time courses for CBNs. **b** Proportion of the indicated cell types in **a**. n = 3 independent experiments; data are presented as mean ± SD. **c** UMAP plots showing expressions of *hrGFP*, *mCherry-WPRE*, and *RPS4Y1* according to scRNA-seq data from differentiation time courses for mCherry-labeled male CBN4 and hrGFP-labeled female CBP9. To minimize batch effects and costs in scRNA-seq, we collected the indicated sample pair as pooled sample by mixing equal cell numbers. **d** UMAP plots of the indicated genes expressed in differentiation time courses for CBN4. PS, primitive streak; PGC, primordial germ cell. **e** IF staining for PGC markers in CBNs on day 2. Arrowheads indicate putative PGC-like cells. **f** Subclustering analysis of scRNA-seq data of Mix cluster in Fig. 3e. UMAP plot is color-coded according to cell subcluster identity annotations. **g** Dot plots of candidate genes specific for the indicated cell subtypes. **h** Pseudotime heatmap showing the top 1500 most variably expressed genes in differentiation time courses for CBNs (top). Temporal sequence schematic of appearance of primitive streak-like intermediates (PSLI) and ExMs (bottom). Color bar denotes relative expression level. Representative genes (left), KEGG (middle) and GO (right) enrichment analysis are shown. Two-sided hypergeometric test, *p*-values were adjusted using the Benjamini-Hochberg method. cP-Value, corrected *p*-value. Scale bars, 100 μm. Source data are provided as a Source Data file.

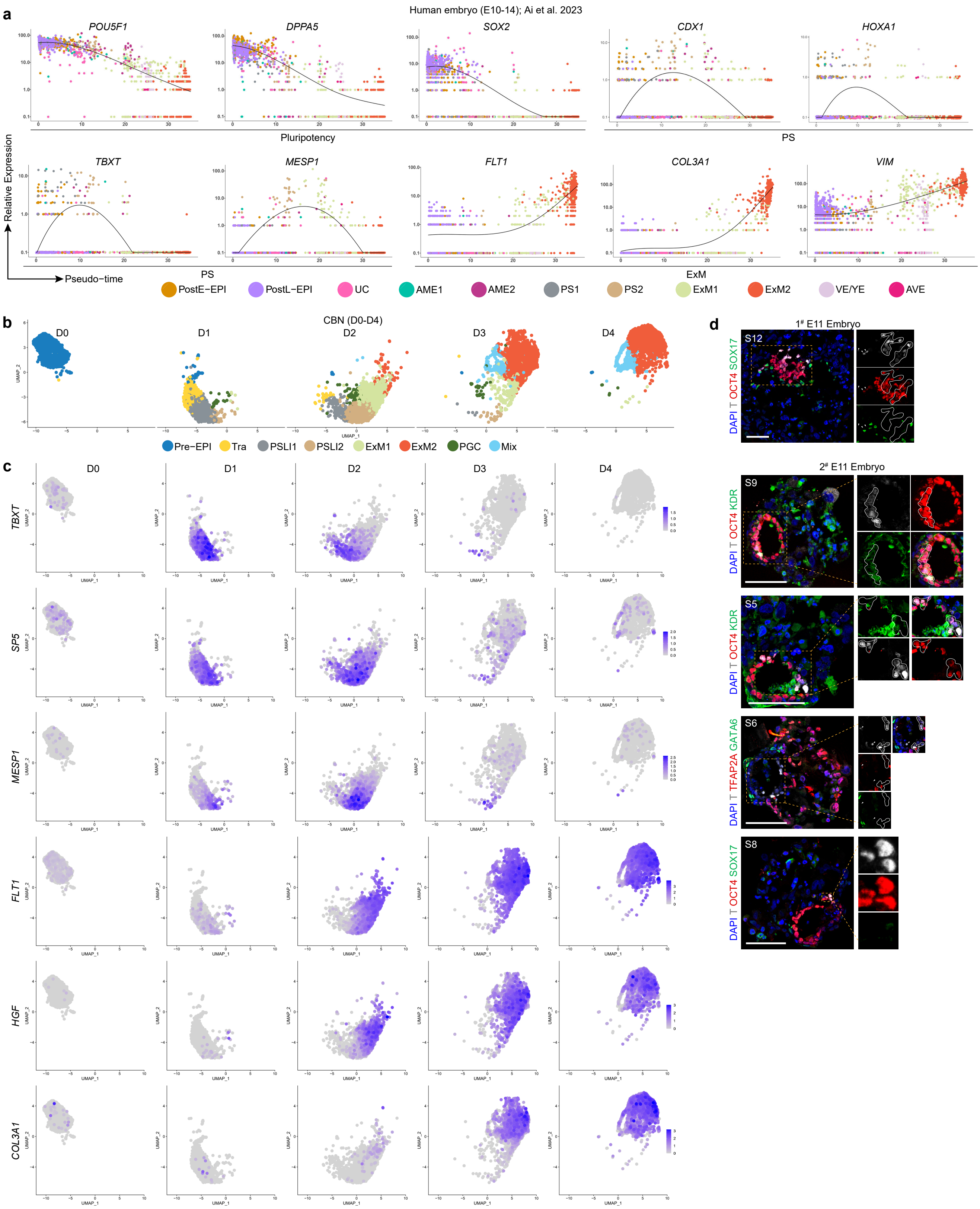
a
Human embryo (E10-14); Ai et al. 2023
POU5F1
DPPA5
SOX2
CDX1
HOXA1
Pluripotency
PS
TBXT
MESP1
FLT1
COL3A1
VIM
PS
ExM
Relative Expression
Pseudo-time
PostE−EPI
PostL−EPI
UC
AME1
AME2
PS1
PS2
ExM1
ExM2
VE/YE
AVE
b
CBN (D0-D4)
D0
D1
D2
D3
D4
Pre−EPI
Tra
PSLI1
PSLI2
ExM1
ExM2
PGC
Mix
c
D0
D1
D2
D3
D4
TBXT
SP5
MESP1
FLT1
HGF
COL3A1
d
1# E11 Embryo
S12
DAPI T OCT4 SOX17
2# E11 Embryo
S9
DAPI T OCT4 KDR
S5
DAPI T OCT4 KDR
S6
DAPI T TFAP2A GATA6
S8
DAPI T OCT4 SOX17

Supplementary Fig. 4 | PSLI specification precedes ExM formation in both cultured human embryos and CBNs, related to Fig. 4. a Dynamic expression of pluripotency, PS, and ExM marker genes in cultured human embryos from embryonic day (E10–14) over pseudotime. Each black line indicates the fitted expression trend of a gene over pseudotime. **b** Split UMAP visualization of scRNA-seq data from differentiation time courses for CBN4. **c** UMAP plots showing the dynamics of the indicated marker genes expressed in differentiation time courses for CBN4. **d** IF staining of the indicated markers in the 1$^{\#}$ (top) and 2$^{\#}$ (bottom) extended cultured human embryos at E11 (see also Fig. 4g). White numbers indicate section (S) numbers, white arrowheads indicate $T^{+}OCT4^{+/weak}SOX17^{–}$ (1$^{\#}$ embryo, S12), $T^{+}OCT4^{–}KDR^{–}$ (2$^{\#}$ embryo, S5), and $T^{+}GATA6^{–}TFAP2A^{–}$ (2$^{\#}$ embryo, S6) PSLIs. White solid circles indicate $T^{+}OCT4^{+/–}SOX17^{–}$ (1$^{\#}$ embryo, S12), $T^{weak}OCT4^{+}KDR^{weak}$ (2$^{\#}$ embryo, S5), $T^{+}OCT4^{+}$ (2$^{\#}$ embryo, S5), and $T^{+}GATA6^{–}TFAP2A^{–}$ (2$^{\#}$ embryo, S6) PSLIs. Red arrowheads indicate $T^{+}OCT4^{+}KDR^{+}$ potential ExM precursors. Scale bars, 100 μm. Source data are provided as a Source Data file.

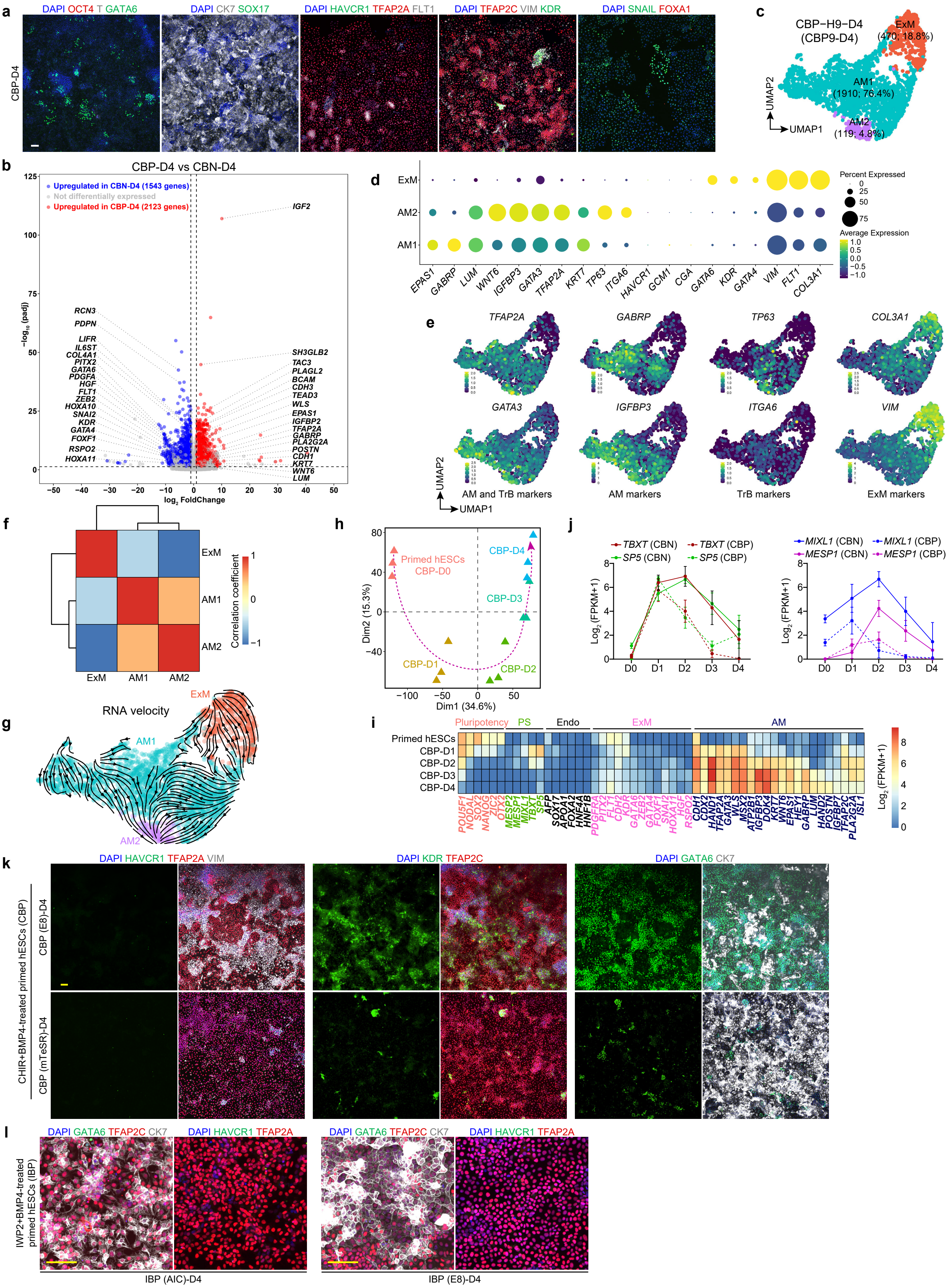

a
DAPI OCT4 T GATA6
DAPI CK7 SOX17
DAPI HAVCR1 TFAP2A FLT1
DAPI TFAP2C VIM KDR
DAPI SNAIL FOXA1
CBP-D4
c
CBP−H9−D4
(CBP9-D4)
ExM
(470; 18.8%)
AM1
(1910; 76.4%)
AM2
(119; 4.8%)
UMAP2
UMAP1
b
CBP-D4 vs CBN-D4
Upregulated in CBN-D4 (1543 genes)
Not differentially expressed
Upregulated in CBP-D4 (2123 genes)
IGF2
RCN3
PDPN
LIFR
IL6ST
COL4A1
PITX2
GATA6
PDGFA
HGF
FLT1
ZEB2
HOXA10
SNAI2
KDR
GATA4
FOXF1
RSPO2
HOXA11
SH3GLB2
TAC3
PLAGL2
BCAM
CDH3
TEAD3
WLS
EPAS1
IGFBP2
TFAP2A
GABRP
PLA2G2A
POSTN
CDH1
KRT7
WNT6
LUM
−log10 (padj)
log2 FoldChange
d
ExM
AM2
AM1
EPAS1
GABRP
LUM
WNT6
IGFBP3
GATA3
TFAP2A
KRT7
TP63
ITGA6
HAVCR1
GCM1
CGA
GATA6
KDR
GATA4
VIM
FLT1
COL3A1
Percent Expressed
Average Expression
e
TFAP2A
GABRP
TP63
COL3A1
GATA3
IGFBP3
ITGA6
VIM
AM and TrB markers
AM markers
TrB markers
ExM markers
UMAP2
UMAP1
f
ExM
AM1
AM2
Correlation coefficient
g
RNA velocity
ExM
AM1
AM2
h
Primed hESCs
CBP-D0
CBP-D4
CBP-D3
CBP-D1
CBP-D2
Dim2 (15.3%)
Dim1 (34.6%)
j
TBXT (CBN)
TBXT (CBP)
SP5 (CBN)
SP5 (CBP)
MIXL1 (CBN)
MIXL1 (CBP)
MESP1 (CBN)
MESP1 (CBP)
Log2 (FPKM+1)
D0
D1
D2
D3
D4
i
Pluripotency
PS
Endo
ExM
AM
Primed hESCs
CBP-D1
CBP-D2
CBP-D3
CBP-D4
Log2 (FPKM+1)
k
DAPI HAVCR1 TFAP2A VIM
DAPI KDR TFAP2C
DAPI GATA6 CK7
CHIR+BMP4-treated primed hESCs (CBP)
CBP (E8)-D4
CBP (mTeSR)-D4
l
DAPI GATA6 TFAP2C CK7
DAPI HAVCR1 TFAP2A
DAPI GATA6 TFAP2C CK7
DAPI HAVCR1 TFAP2A
IWP2+BMP4-treated primed hESCs (IBP)
IBP (AIC)-D4
IBP (E8)-D4

Supplementary Fig. 5 | Primed hESCs respond differently to CB conditions compared to naive hESCs, related to Fig. 5. a IF staining for the indicated embryonic and extraembryonic lineage markers in CB-treated primed hESCs on day 4 (CBP-D4). **b** Volcano plot representing the differentially expressed genes between CBP-D4 and CBN-D4, $\log_2$ FC ≥ 1 or ≤ -1, padj < 0.05. Two-sided Wald test, *p*-values were adjusted using the Benjamini-Hochberg method. **c** UMAP visualization of single-cell transcriptomics from CBP9-D4. UMAP plot is color-coded according to cell cluster identity annotations. **d** Dot plots of candidate genes specific for cell subtypes. **e** UMAP plots of the indicated genes expressed in CBP9-D4. **f** Spearman correlation heatmap of distinct cell subpopulations, the colors indicate the Spearman correlation coefficient. **g** RNA velocity vectors projected onto the UMAP-based embeddings of the scRNA-seq dataset shown in **c**. **h** PCA of bulk RNA-seq data from differentiation time courses for CBPs, computed using the genes with FPKM ≥ 1 in at least one sample. **i** Heatmap of representative pluripotency and lineage marker genes in differentiation time courses for CBPs. **j** Expression dynamics of PS marker genes in differentiation time courses for CBNs and CBPs. n = 3 hESC lines; data are presented as mean ± SD. **k** IF staining for the indicated markers in D4 CBPs, primed hESCs were cultured in E8 (top) or mTeSR1 (bottom) medium (unless otherwise specified, primed hESCs were typically cultured in AIC medium). **l** IF staining for the indicated markers in IWP2+BMP4-treated primed hESCs on day 4 (IBP-D4), primed hESCs were cultured in AIC (left) or E8 (right) medium. Scale bars, 100 μm. Source data are provided as a Source Data file.

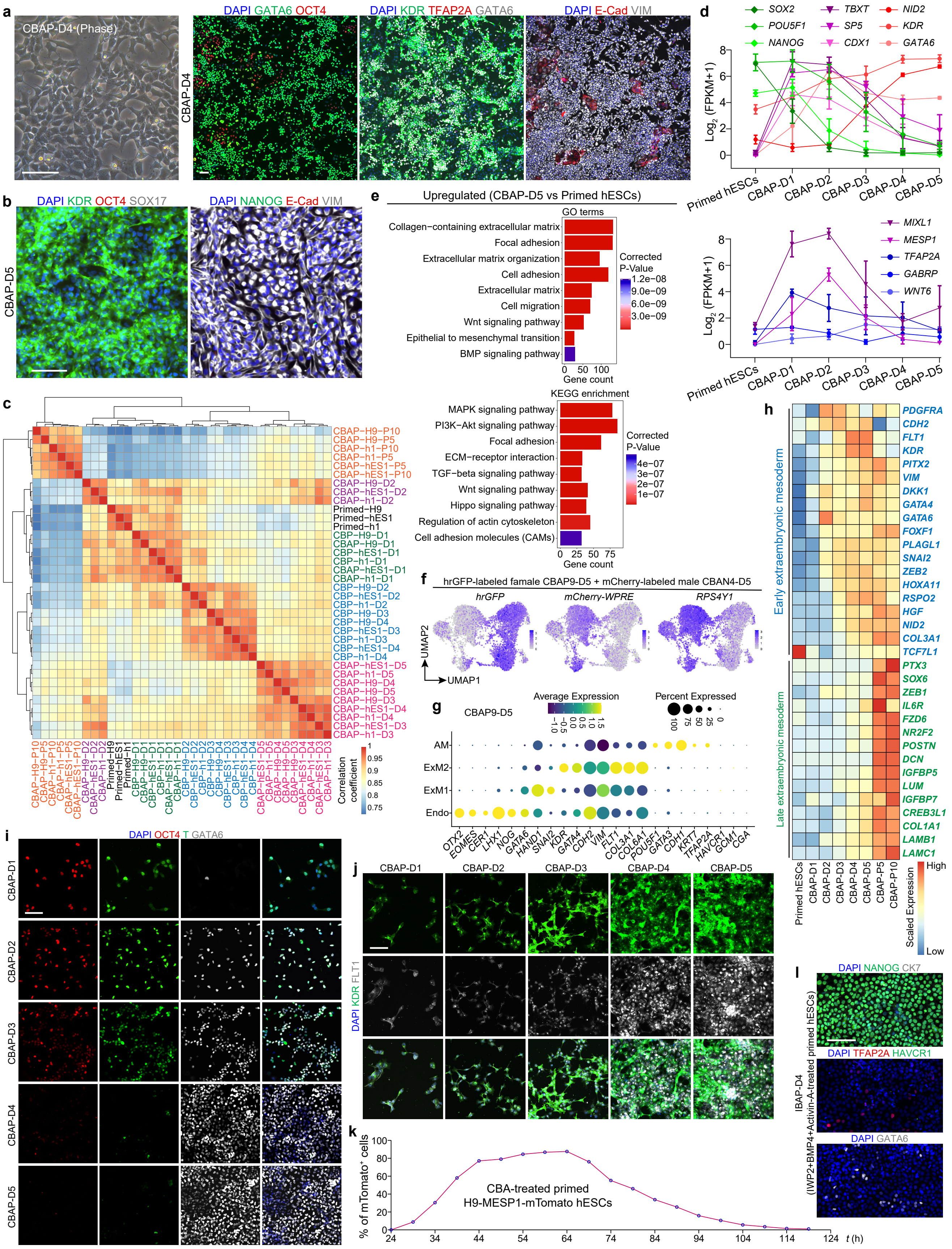
a
CBAP-D4 (Phase)
DAPI GATA6 OCT4
DAPI KDR TFAP2A GATA6
DAPI E-Cad VIM
CBAP-D4
b
DAPI KDR OCT4 SOX17
DAPI NANOG E-Cad VIM
CBAP-D5
c
d
SOX2
POU5F1
NANOG
TBXT
SP5
CDX1
NID2
KDR
GATA6
Log2 (FPKM+1)
Primed hESCs
CBAP-D1
CBAP-D2
CBAP-D3
CBAP-D4
CBAP-D5
MIXL1
MESP1
TFAP2A
GABRP
WNT6
e
Upregulated (CBAP-D5 vs Primed hESCs)
GO terms
Collagen−containing extracellular matrix
Focal adhesion
Extracellular matrix organization
Cell adhesion
Extracellular matrix
Cell migration
Wnt signaling pathway
Epithelial to mesenchymal transition
BMP signaling pathway
Corrected P-Value
Gene count
KEGG enrichment
MAPK signaling pathway
PI3K−Akt signaling pathway
Focal adhesion
ECM−receptor interaction
TGF−beta signaling pathway
Wnt signaling pathway
Hippo signaling pathway
Regulation of actin cytoskeleton
Cell adhesion molecules (CAMs)
f
hrGFP-labeled famale CBAP9-D5 + mCherry-labeled male CBAN4-D5
hrGFP
mCherry-WPRE
RPS4Y1
UMAP1
UMAP2
g
CBAP9-D5
Average Expression
Percent Expressed
AM
ExM2
ExM1
Endo
h
Early extraembryonic mesoderm
Late extraembryonic mesoderm
Scaled Expression
High
Low
i
DAPI OCT4 T GATA6
CBAP-D1
CBAP-D2
CBAP-D3
CBAP-D4
CBAP-D5
j
DAPI KDR FLT1
k
% of mTomato+ cells
CBA-treated primed H9-MESP1-mTomato hESCs
t (h)
l
DAPI NANOG CK7
DAPI TFAP2A HAVCR1
DAPI GATA6
IBAP-D4 (IWP2+BMP4+Activin-A-treated primed hESCs)

Supplementary Fig. 6 | Primed hESCs efficiently differentiate into ExMs via PSLIs under CBA conditions, related to Fig. 6. a Representative brightfield and IF staining images for the indicated markers in D4 CBAPs. **b** Representative IF staining images for the indicated markers in D5 CBAPs. **c** Spearman correlation analysis of gene expression patterns in differentiation time courses for primed hESCs under CBP and CBAP conditions. **d** Expression dynamics of the indicated marker genes in differentiation time courses for CBAPs. n = 3 hESC lines; data are presented as mean ± SD. **e** GO and KEGG analyses showing upregulated gene categories in D5 CBAPs relative to primed hESCs. Two-sided hypergeometric test, *p*-values were adjusted using the Benjamini-Hochberg method. **f** UMAP plots showing expressions of *hrGFP*, *mCherry-WPRE*, and Y-chromosome specific gene *RPS4Y1* according to scRNA-seq data from CBA-treated primed H9 (CBAP9-D5, hrGFP-labeled) and AIC-N4 (CBAN4-D5, mCherry-labeled) hESCs on day 5. To minimize batch effects and costs in scRNA-seq, we collected the indicated sample pair as pooled sample by mixing equal cell numbers. **g** Dot plots of candidate genes specific for cell subtypes in CBAP9-D5. **h** Heatmap of representative early and late ExM marker genes in differentiation time courses for CBAPs. Values represent $\log_2$ (FPKM+1) scaled by gene expression across samples. **i**, **j** IF staining to demonstrate the dynamic expression of the indicated markers in differentiation time courses for CBAPs. **k** The dynamics of the proportion of mTomato$^+$ cells in the differentiation time courses for MESP1-mTomato knock-in reporter CBAPs, constructed using data from Supplementary Movies 1, 2. **l** IF staining for the indicated markers in IWP2+BMP4+Activin-A-treated primed hESCs on day 4 (IBAP-D4). Scale bars, 100 μm. Source data are provided as a Source Data file.

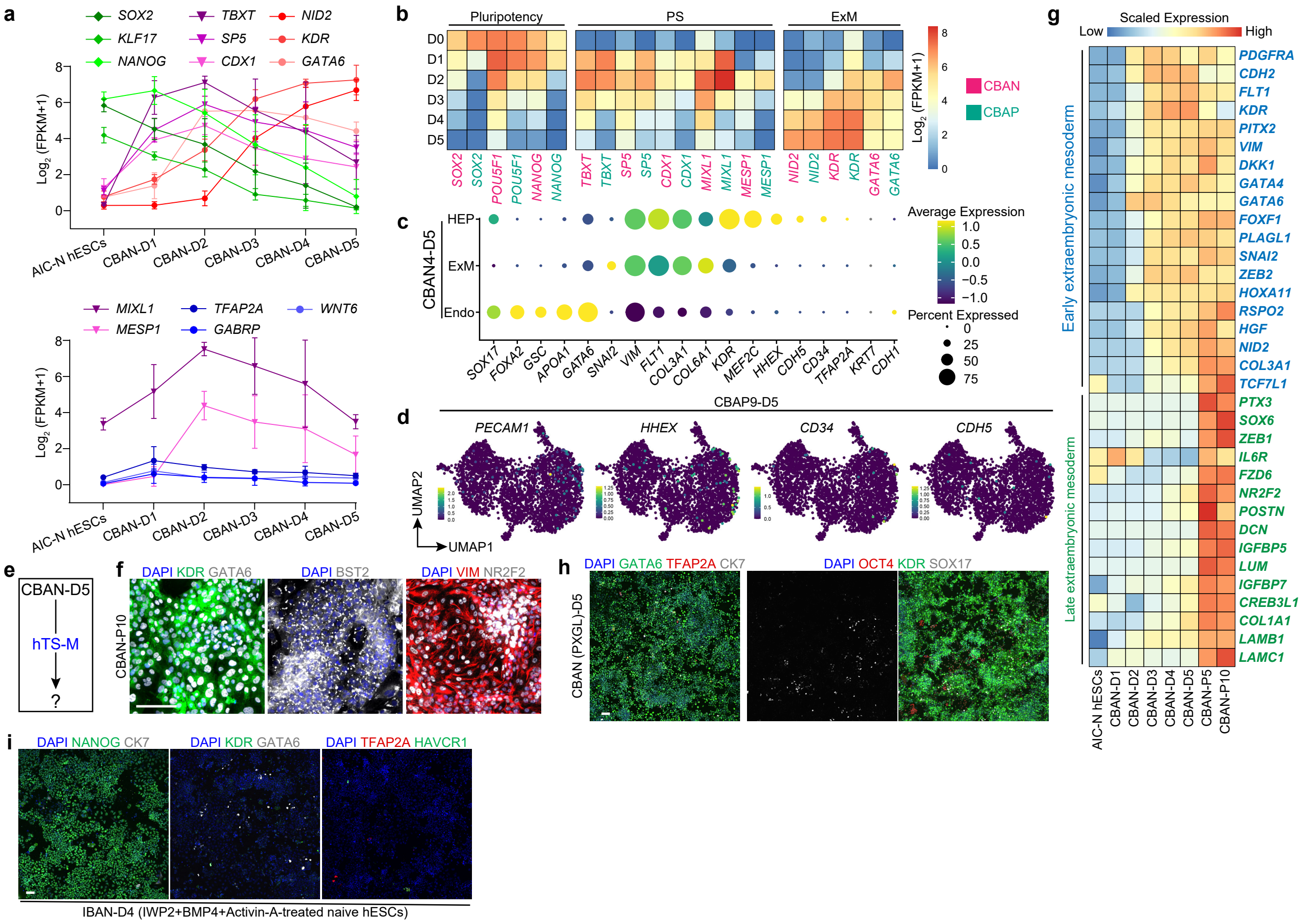

a
SOX2
KLF17
NANOG
TBXT
SP5
CDX1
NID2
KDR
GATA6
Log2 (FPKM+1)
AIC-N hESCs
CBAN-D1
CBAN-D2
CBAN-D3
CBAN-D4
CBAN-D5
MIXL1
MESP1
TFAP2A
GABRP
WNT6
b
Pluripotency
PS
ExM
D0
D1
D2
D3
D4
D5
CBAN
CBAP
c
CBAN4-D5
HEP
ExM
Endo
Average Expression
Percent Expressed
SOX17
FOXA2
GSC
APOA1
GATA6
SNAI2
VIM
FLT1
COL3A1
COL6A1
KDR
MEF2C
HHEX
CDH5
CD34
TFAP2A
KRT7
CDH1
d
CBAP9-D5
PECAM1
HHEX
CD34
CDH5
UMAP1
UMAP2
e
CBAN-D5
hTS-M
?
f
CBAN-P10
DAPI KDR GATA6
DAPI BST2
DAPI VIM NR2F2
g
Scaled Expression
Low
High
Early extraembryonic mesoderm
Late extraembryonic mesoderm
PDGFRA
CDH2
FLT1
KDR
PITX2
VIM
DKK1
GATA4
GATA6
FOXF1
PLAGL1
SNAI2
ZEB2
HOXA11
RSPO2
HGF
NID2
COL3A1
TCF7L1
PTX3
SOX6
ZEB1
IL6R
FZD6
NR2F2
POSTN
DCN
IGFBP5
LUM
IGFBP7
CREB3L1
COL1A1
LAMB1
LAMC1
AIC-N hESCs
CBAN-D1
CBAN-D2
CBAN-D3
CBAN-D4
CBAN-D5
CBAN-P5
CBAN-P10
h
CBAN (PXGL)-D5
DAPI GATA6 TFAP2A CK7
DAPI OCT4 KDR SOX17
i
DAPI NANOG CK7
DAPI KDR GATA6
DAPI TFAP2A HAVCR1
IBAN-D4 (IWP2+BMP4+Activin-A-treated naive hESCs)

Supplementary Fig. 7 | Naive hESCs efficiently differentiate into ExMs via PSLIs under CBA conditions, related to Fig. 7. a Expression dynamics of the indicated marker genes during differentiation time courses for CBANs. n = 3 hESC lines; data are presented as mean ± SD. **b** Heatmap of representative pluripotency, PS, and ExM marker genes during differentiation time courses for CBANs and CBAPs. **c** Dot plots of candidate genes specific for cell subtypes in CBAN4-D5. Endo, endoderm. **d** UMAP plots of the hematopoietic marker genes expressed in CBAP9-D5. **e** Schematic of CBAN-D5 inoculated into hTS-M for extended culture. **f** IF staining images for the indicated markers in expandable CBANs at passage 10. **g** Heatmap depicting representative early and late ExM marker gene expression across differentiation time courses for CBANs. Values represent $\log_2$ (FPKM+1) scaled by gene expression across samples. **h** IF staining images for the indicated markers in CBAN-D5, naive hESCs were cultured in PXGL medium. **i** IF staining for the indicated markers in IWP2+BMP4+Activin-A-treated naive hESCs on day 4 (IBAN-D4). Scale bars, 100 μm. Source data are provided as a Source Data file.

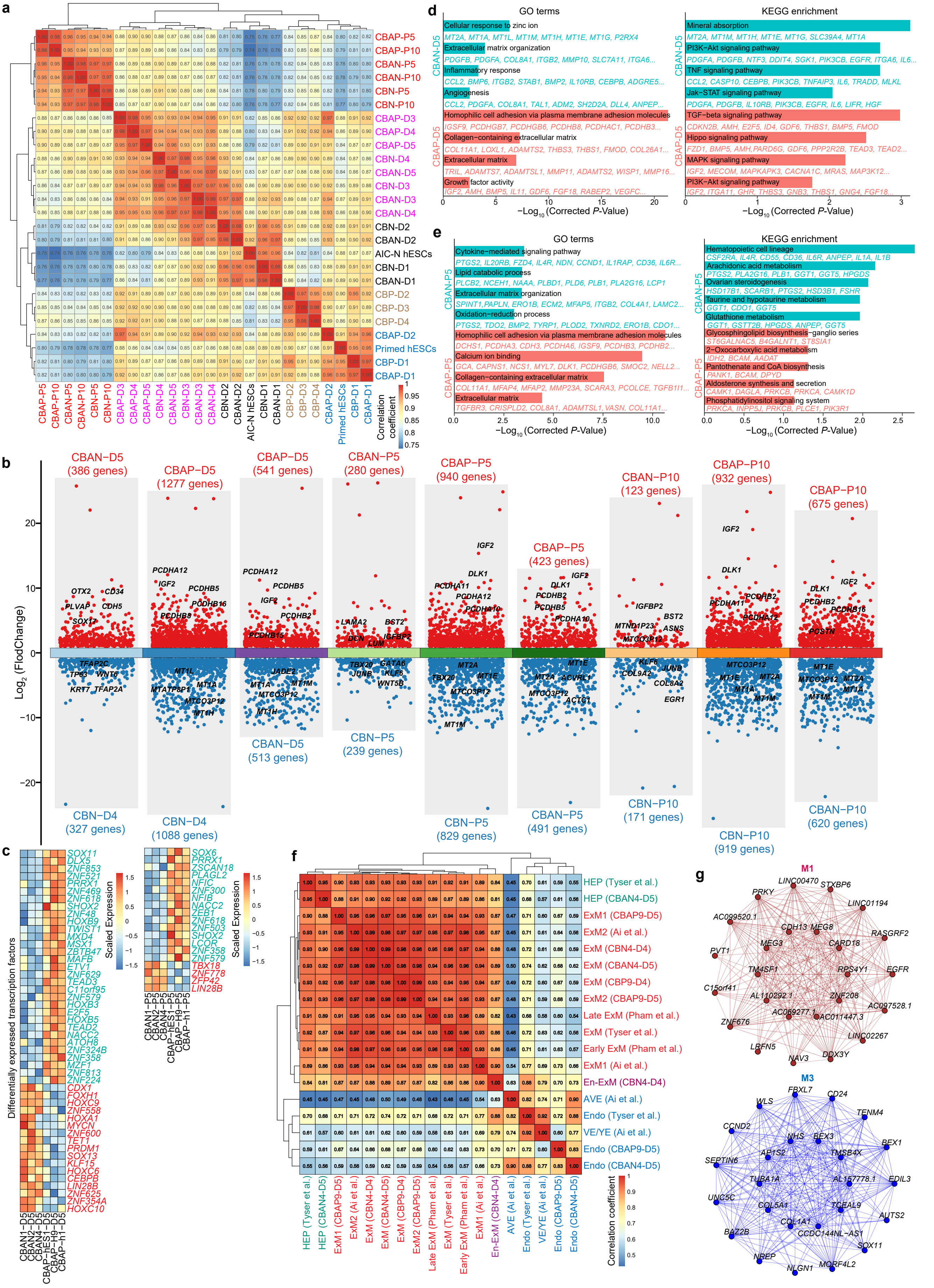
a
CBAP-P5
CBAP-P10
CBAN-P5
CBAN-P10
CBN-P5
CBN-P10
CBAP-D3
CBAP-D4
CBAP-D5
CBN-D4
CBAN-D5
CBN-D3
CBAN-D3
CBAN-D4
CBN-D2
CBAN-D2
AIC-N hESCs
CBN-D1
CBAN-D1
CBP-D2
CBP-D3
CBP-D4
CBAP-D2
Primed hESCs
CBP-D1
CBAP-D1
Correlation coefficient
b
Log2 (FlodChange)
CBAN-D5 (386 genes)
CBAP-D5 (1277 genes)
CBAP-D5 (541 genes)
CBAN-P5 (280 genes)
CBAP-P5 (940 genes)
CBAP-P5 (423 genes)
CBAN-P10 (123 genes)
CBAP-P10 (932 genes)
CBAP-P10 (675 genes)
CBN-D4 (327 genes)
CBN-D4 (1088 genes)
CBAN-D5 (513 genes)
CBN-P5 (239 genes)
CBN-P5 (829 genes)
CBAN-P5 (491 genes)
CBN-P10 (171 genes)
CBN-P10 (919 genes)
CBAN-P10 (620 genes)
c
Differentially expressed transcription factors
Scaled Expression
CBAN1-D5
CBAN2-D5
CBAN4-D5
CBAP-hES1-D5
CBAP-H9-D5
CBAP-h1-D5
CBAN1-P5
CBAN2-P5
CBAN4-P5
CBAP-hES1-P5
CBAP-H9-P5
CBAP-h1-P5
d
GO terms
KEGG enrichment
CBAN-D5
CBAP-D5
Cellular response to zinc ion
Extracellular matrix organization
Inflammatory response
Angiogenesis
Homophilic cell adhesion via plasma membrane adhesion molecules
Collagen−containing extracellular matrix
Extracellular matrix
Growth factor activity
Mineral absorption
PI3K−Akt signaling pathway
TNF signaling pathway
Jak−STAT signaling pathway
TGF−beta signaling pathway
Hippo signaling pathway
MAPK signaling pathway
PI3K−Akt signaling pathway
−Log10 (Corrected P-Value)
e
GO terms
KEGG enrichment
CBAN-P5
CBAP-P5
Cytokine−mediated signaling pathway
Lipid catabolic process
Extracellular matrix organization
Oxidation−reduction process
Homophilic cell adhesion via plasma membrane adhesion molecules
Calcium ion binding
Collagen−containing extracellular matrix
Extracellular matrix
Hematopoietic cell lineage
Arachidonic acid metabolism
Ovarian steroidogenesis
Taurine and hypotaurine metabolism
Glutathione metabolism
Glycosphingolipid biosynthesis−ganglio series
2−Oxocarboxylic acid metabolism
Pantothenate and CoA biosynthesis
Aldosterone synthesis and secretion
Phosphatidylinositol signaling system
−Log10 (Corrected P-Value)
f
HEP (Tyser et al.)
HEP (CBAN4-D5)
ExM1 (CBAP9-D5)
ExM2 (Ai et al.)
ExM (CBN4-D4)
ExM (CBAN4-D5)
ExM (CBP9-D4)
ExM2 (CBAP9-D5)
Late ExM (Pham et al.)
ExM (Tyser et al.)
Early ExM (Pham et al.)
ExM1 (Ai et al.)
En-ExM (CBN4-D4)
AVE (Ai et al.)
Endo (Tyser et al.)
VE/YE (Ai et al.)
Endo (CBAP9-D5)
Endo (CBAN4-D5)
Correlation coefficient
g
M1
M3

Supplementary Fig. 8 | Naive and primed hESC-derived ExMs exhibit both shared and distinct transcriptional characteristics, related to Fig. 8. a Spearman correlation analysis of gene expression patterns during differentiation time courses for naive and primed hESCs under CBP and CBAP conditions. **b** Differential gene expression analysis across nine different comparison groups. p-adj < 0.05 and the y-axis represents the $\log_2$ fold change. Two-sided Wald test, p-values were adjusted using the Benjamini-Hochberg method. **c** Heatmap of the differentially expressed transcription factors between CBANs and CBAPs at different time points (D5/P5). Log_2 (FC) ≥ 1, p-adj < 0.05, and FPKM ≥ 3 in at least two samples. Values represent $\log_2$ (FPKM+1) scaled by gene expression across samples. Two-sided Wald test, p-values were adjusted using the Benjamini-Hochberg method. **d**, **e** Bar plots displaying the GO and KEGG analyses of differentially expressed genes between CBANs and CBAPs on D5 (**d**) and at P5 (**e**). Two-sided hypergeometric test, p-values were adjusted using the Benjamini-Hochberg method. **f** Spearman correlation heatmap of different cell clusters, the colors indicate the Spearman correlation coefficient. **g** Co-expression network plots for M1 and M3 in Fig. 8h. M, module. Source data are provided as a Source Data file.

Supplementary Table 1: A list of antibodies used in this study

Antibody information for flow cytometry

**Antibody (species/isotype)**	**Source**	**Cat#**	**Lot**	**Dilution**
GATA6 (D61E4) XP® Rabbit mAb (PE Conjugate)	Cell Signaling Technology	26452	4	1:600
Rabbit (DA1E) mAb IgG XP® Isotype Control (PE Conjugate)	Cell Signaling Technology	5742	17	1:600
Goat Anti-Human SNAIL	R&D Systems	AF-3639	XRS0217071	1:400
Goat IgG Isotype Control	Thermo Fisher Scientific	31245	0960621	1:400
APC anti-human CD324 (E-Cadherin) Antibody	Biolegend	324108	B379257	1:100
APC Mouse IgG1, κ Isotype Ctrl (FC) Antibody	Biolegend	400122	B379803	1:100
Recombinant Anti-Transcription factor AP-2-alpha antibody [EPR2688(2)]	Abcam	ab108311	1014077-1	1:200
Recombinant Rabbit IgG, monoclonal [EPR25A] - Isotype Control	Abcam	ab172730	1011223-6	1:200
Alexa Fluor 555 donkey anti-rabbit IgG	Thermo Fisher Scientific	A-31572	1866859	1:1000
Alexa Fluor 488 donkey anti-goat IgG	Thermo Fisher Scientific	A-11055	1827671	1:1000

Antibody information for immunofluorescence staining

**Antibody (species/isotype)**	**Source**	**Cat#**	**Lot#**	**Dilution**
NANOG (goat IgG)	R&D Systems	AF1997	KKJ0922031	1:400
OCT4 (mouse IgG)	Santa Cruz	sc-5279	C3121	1:600
BLIMP1 (rabbit IgG)	Cell Signaling Technology	9115S	6	1:100
MEIS2 (mouse IgM)	Novus Biologicals	H00004212-M01	M3011-EH4	1:200
E-Cadherin (mouse IgG)	Abcam	ab76055	GR3360021-7	1:200
BST2 (rabbit IgG)	Abcam	ab243230	1022489-8	1:200
Active β-Catenin (rabbit IgG)	Cell Signaling Technology	19807S	4	1:400
Phospho-Smad1/Smad5/Smad9 (rabbit IgG)	Cell Signaling Technology	13820S	3	1:400
GATA6 (goat IgG)	R&D Systems	AF1700	KWT0523031	1:600
GATA6 (rabbit IgG)	Cell Signaling Technology	5851S	5	1:600
SOX17 (goat IgG)	R&D Systems	AF1924	KGA08150422	1:600
SOX17 (rabbit IgG)	Cell Signaling Technology	81778S	1	1:500
CK7/KRT7 (rabbit IgG)	Abcam	ab181598	GR3321316-17	1:500
NR2F2 (rabbit IgG)	Abcam	ab211776	1006695-11	1:200
TFAP2C (mouse IgG)	Santa Cruz	sc-12762	D2717	1:400
Brachyury/T (goat IgG)	R&D Systems	AF2085	KQP0719121	1:400
Brachyury/T (rabbit IgG)	Cell Signaling Technology	81694S	1	1:800
HAVCR1 (goat IgG)	R&D Systems	AF1750	GTB0618111	1:200
TFAP2A (mouse IgG)	Santa Cruz	sc-12726	L2619	1:400

SNAIL (goat IgG)	R&D Systems	AF-3639	XRS0217071	1:400
VIMENTIN (mouse IgG)	eBioscience	14-9897	2297463	1:2000
VIMENTIN (rabbit IgG)	Abcam	ab137321	GR3425580-7	1:1000
CD34 (rabbit IgG)	Abcam	ab81289	1071545-11	1:100
CD31 (mouse IgG)	Abcam	ab9498	1035700-12	1:100
LUM (rabbit IgG)	Invitrogen	MA5-29402	35FC7A02	1:100
DCN (rabbit IgG)	Abcam	ab151988	GR122269-56	1:100
FLT1 (rabbit IgG)	Abcam	ab32152	GR3321125-17	1:200
FOXA1 (mouse IgG)	Abcam	ab55178	GR3448710-4	1:100
KDR (goat IgG)	R&D Systems	AF357	CUE0620061	1:400
Alexa Fluor 488 donkey anti-rabbit IgG	Jackson Immunoresearch	711-545-152	139808	1:600
Alexa Fluor 568 donkey anti-mouse IgG	Thermo Fisher Scientific	A-10037	1827879	1:600
Alexa Fluor 488 donkey anti-mouse IgG	Thermo Fisher Scientific	A-21202	1820538	1:600
Alexa Fluor 647 donkey anti-rabbit IgG	Thermo Fisher Scientific	A-31573	1874788	1:600
Alexa Fluor 555 donkey anti-rabbit IgG	Thermo Fisher Scientific	A-31572	1866859	1:600
Alexa Fluor 647 donkey anti-goat IgG	Thermo Fisher Scientific	A-21447	1841382	1:600
Alexa Fluor 488 donkey anti-goat IgG	Thermo Fisher Scientific	A-11055	1827671	1:600
