# Supplementary material for: Deciphering signaling mechanisms and developmental dynamics in extraembryonic mesoderm specification from hESCs
Source: Nat Commun. 2025 May 21;16:4688. doi: 10.1038/s41467-025-59491-x (PMC12095623; doi:10.1038/s41467-025-59491-x)
Supplement: Supplementary file 2 — Description of Additional Supplementary Files [file 41467_2025_59491_MOESM2_ESM.pdf]

Description of Additional Supplementary Files

File Name: Supplementary Movie 1
Description: Live-cell imaging showing the expression dynamics of mTomato in the differentiation time courses for MESP1-mTomato knock-in reporter hESCs under CBA conditions (Merge bright-field and fluorescence).

File Name: Supplementary Movie 2
Description: Live-cell imaging showing the expression dynamics of mTomato in the differentiation time courses for MESP1-mTomato knock-in reporter hESCs under CBA conditions (Split bright-field and fluorescence).
