# Supplementary material for: Deciphering signaling mechanisms and developmental dynamics in extraembryonic mesoderm specification from hESCs
Source: Nat Commun. 2025 May 21;16:4688. doi: 10.1038/s41467-025-59491-x (PMC12095623; doi:10.1038/s41467-025-59491-x)
Supplement: Supplementary file 5 — Reporting Summary [file 41467_2025_59491_MOESM5_ESM.pdf]

nature portfolio

Corresponding author(s): Zongyong Ai

Last updated by author(s): Apr 7, 2025

Reporting Summary

Nature Portfolio wishes to improve the reproducibility of the work that we publish. This form provides structure for consistency and transparency in reporting. For further information on Nature Portfolio policies, see our Editorial Policies and the Editorial Policy Checklist.

Statistics

For all statistical analyses, confirm that the following items are present in the figure legend, table legend, main text, or Methods section.

n/a	Confirmed	
☐	☒	The exact sample size (*n*) for each experimental group/condition, given as a discrete number and unit of measurement
☐	☒	A statement on whether measurements were taken from distinct samples or whether the same sample was measured repeatedly
☐	☒	The statistical test(s) used AND whether they are one- or two-sided<br>*Only common tests should be described solely by name; describe more complex techniques in the Methods section.*
☒	☐	A description of all covariates tested
☒	☐	A description of any assumptions or corrections, such as tests of normality and adjustment for multiple comparisons
☐	☒	A full description of the statistical parameters including central tendency (e.g. means) or other basic estimates (e.g. regression coefficient) AND variation (e.g. standard deviation) or associated estimates of uncertainty (e.g. confidence intervals)
☐	☒	For null hypothesis testing, the test statistic (e.g. *F*, *t*, *r*) with confidence intervals, effect sizes, degrees of freedom and *P* value noted<br>*Give P values as exact values whenever suitable.*
☒	☐	For Bayesian analysis, information on the choice of priors and Markov chain Monte Carlo settings
☒	☐	For hierarchical and complex designs, identification of the appropriate level for tests and full reporting of outcomes
☒	☐	Estimates of effect sizes (e.g. Cohen's *d*, Pearson's *r*), indicating how they were calculated

Our web collection on statistics for biologists contains articles on many of the points above.

Software and code

Policy information about availability of computer code

Data collection	Microscopy images were taken by Nikon AX Microsystems, Leica SP8 Microsystems and Yokogawa CellVoyager CQ1 Benchtop. The Flow cytometry data were collected by FACSAria III. RNA-Sequencing, 10x Genomics scRNA-sequencing were performed at Annoroad Gene Technology (http://www.annoroad.com/).
Data analysis	Images were processed with Leica software (LAS X) and NIS-Elements Viewer (V5.21) and Imaris (V10.0) and Fiji (V1.54). Flow cytometry data were processed Flow Jo software (V10.6.2). Microsoft Excel (2021) and Graphpad Prism (V10.0) were used to analyze statistical and draw graphs.<br>The fluorescence intensity was analyzed using Fiji software, and the mean fluorescence intensities of individual cells were taken as the total fluorescence intensity within the cell region divided by the area of that cell. The fluorescence intensity profile was generated using the Line Plot function in FIJI software: (1) draw a line using the Line tool; (2) run the command Analyze > Plot Profile to generate an intensity line plot; and (3) click List to display and export the plot values. The mean fluorescence intensities were measured using FIJI software: (1) select the region and adjust the threshold through "Image-Adjust-Threshold"; (2) set the parameters through "Analyze-Set Measurements"; and (3) run "analytic-measure" and export the plot values. To quantify the proportions of different cell types via immunofluorescence, at least three fields were randomly captured using a confocal microscope with a 10× objective lens for each experiment. Total cell numbers (DAPI-stained nuclei) and the numbers of different cell types (expressing specific markers) were quantified using the spots tool in Imaris software (version 10.0, Oxford Instruments). The graphs were plotted using GraphPad Prism 9.0.<br>Reads were aligned to human genome (UCSC GRCh38) using HISAT2 (v2.2.1)44. The counts and FPKM values for each gene were calculated with StringTie (v2.1.1)45. Principal components analysis was performed using prcomp function from the R stats package based on the gene matrix with FPKM ≥ 1 in at least one sample. Heatmaps were generated using pheatmap package from the R software.<br>Correlation analysis was performed by Spearman correlation based on the gene matrix with FPKM ≥ 1 in at least one sample. Differentially expressed genes were detected by the package DESeq2 (v1.42.1)46 in the R software. An adjusted p-value < 0.05 and an absolute value of the

log2 Fold Change ≥ 1 and FPKM ≥ 1 in at least one sample were used as the threshold for declaring gene expression differences as being significant. Gene Ontology (GO) and Kyoto Encyclopedia of Genes and Genomes (KEGG) analyses were conducted with the KOBAS (http://bioinfo.org/kobas).Sequencing data was aligned and quantified using the Cell Ranger Pipeline v7.0.1 (10x Genomics) against the GRCh38 reference genome with hrGFP and mCherry-WPRE. scRNA-seq data were filtered based on number of expressed genes and expression level of mitochondrial genes (below 15%). Cell doublets were removed by DoubletFinder (v2.0.3) with assuming multiplet rate according to the loaded cells number (refer to Multiplet Rate Table provided in the 10x Genomics User Guide). Gene expression levels (normalized and natural-log (log1p) transformed value) of RPS4Y1, hrGFP, and mCherry-WPRE were used to determine the source of different cell types in pooled samples. Cells positive for mCherry-WPRE (value > 0) and negative for hrGFP (value = 0) were defined as derivatives from the mCherry-labeled male AIC-N4 hESCs and further filtered with RPS4Y1 expression (value > 1), and cells positive for hrGFP (value > 0) and negative for mCherry-WPRE (value = 0) were defined as derivatives from hrGFP-labeled female primed H9 hESCs and further filtered with RPS4Y1 expression (value < 1). Double positive or double negative cells were removed for further analyses.Further analyses were performed using Seurat package (v4.3.0.1). The raw counts were normalized and scaled with default parameters. Top 2000 most variable genes were identified and used for dimensionality reduction with PCA and followed with non-linear dimensionality reduction using UMAP. The scRNA-seq data from differentiation time courses for CBNs or CBPs were combined with merge() function. Cell types were defined based on the lineage markers and clusters identified through FindClusters() function. Data was visualized with the UMAP dimensionality reduction. Differentially expressed genes were identified with the FindAllMarkers() function in Seurat and filtered with Padj of Wilcoxon's rank-sum test < 0.05, log2 (FC)>0.25 and expressed in > 25% of cells of the given cluster. GO and KEGG analyses were conducted with the KOBAS (http://bioinfo.org/kobas). Single-cell pseudotime trajectories were constructed with Monocle 2 (v2.18.0) and Slingshot (v1.8.0). RNA velocity analysis was performed by scVelo (v0.3.1).

For manuscripts utilizing custom algorithms or software that are central to the research but not yet described in published literature, software must be made available to editors and reviewers. We strongly encourage code deposition in a community repository (e.g. GitHub). See the Nature Portfolio guidelines for submitting code & software for further information.

Data

Policy information about availability of data

All manuscripts must include a data availability statement. This statement should provide the following information, where applicable:

- Accession codes, unique identifiers, or web links for publicly available datasets
- A description of any restrictions on data availability
- For clinical datasets or third party data, please ensure that the statement adheres to our policy

The raw sequence data from our study have been deposited in the Genome Sequence Archive in National Genomics Data Center (https://www.cncb.ac.cn/) under the BioProject accession code PRJCA037022. This paper does not report original code, publicly available tools were used in data analysis as described. Any additional information required to reanalyze the data reported in this paper is available from the lead contact upon request. Previously publsihed datasets were available: bulk RNA-seq datasets from primed hESCs (GEO, GSE145163); bulk RNA-seq datasets from naive hESCs and scRNA-seq datasets from cultured human embryos (NGDC, https://ngdc.cncb.ac.cn/, PRJCA017779); scRNA-seq datasets from human CS7 gastrulating embryo (GEO, GSE193007); and scRNA-seq datasets from naive hESC derivatives in hTS-M ASECRiAV (GEO, GSE204779).

Research involving human participants, their data, or biological material

Policy information about studies with human participants or human data. See also policy information about sex, gender (identity/presentation), and sexual orientation and race, ethnicity and racism.

Reporting on sex and gender	Frozen embryos were donated to research. The sex of the embryos cultured in the lab is largely unknown.
Reporting on race, ethnicity, or other socially relevant groupings	Donors are couples who have received help conceiving with IVF in Department of Reproductive Medicine, The First People's Hospital of Yunnan Province, Kunming, Yunnan, China. Race or ethnicity of the donors are unknown.
Population characteristics	The population characteristics of the donating patients and their embryos are unknown.
Recruitment	All human embryos used were donated by couples who had completed their reproductive goals following IVF treatment. Written informed consent was obtained from both partners of each couple. No financial incentives were offered for the donation.
Ethics oversight	The research about human embryo is a continuation of our previous work and had been approved by the Medicine Ethics Committee of The First People's Hospital of Yunnan Province (KHLL2020-KY064)3,25. All donated embryos were supernumerary frozen embryos after IVF clinic treatment. The informed consent process for embryo donation complied with International Society for Stem Cell Research (ISSCR) Guidelines (2021) and Ethical Guidelines for Human Embryonic Stem Cell research (2003) jointly issued by Ministry of Science and Technology and Ministry of Health of People's Republic of China. All the donor couples had signed the informed consent for voluntary donations of supernumerary embryos for this study. No financial inducements were offered for the donations.

Note that full information on the approval of the study protocol must also be provided in the manuscript.

Field-specific reporting

Please select the one below that is the best fit for your research. If you are not sure, read the appropriate sections before making your selection.

☒ Life sciences ☐ Behavioural & social sciences ☐ Ecological, evolutionary & environmental sciences

Life sciences study design

All studies must disclose on these points even when the disclosure is negative.

Sample size	Sample sizes and p values were provided in figure legends or the Source Data. Statistical analyses were performed with three or more than three independent experimental replicates.
Data exclusions	No data were excluded from the analysis.
Replication	The number of replicated experiments were provided in methods or figure legends. All the attempts at replication were successful.
Randomization	No randomization was performed. we were differentiating cells batch by batch, data were collected from all batches.
Blinding	The investigators were not blinded to allocation during experiments and outcome assessment. Data collection and analysis were performed by different people, the sample classification were replaced by simple marks during data analysis.

Reporting for specific materials, systems and methods

We require information from authors about some types of materials, experimental systems and methods used in many studies. Here, indicate whether each material, system or method listed is relevant to your study. If you are not sure if a list item applies to your research, read the appropriate section before selecting a response.

Materials & experimental systems

n/a	Involved in the study	
☐	☒	Antibodies
☐	☒	Eukaryotic cell lines
☒	☐	Palaeontology and archaeology
☒	☐	Animals and other organisms
☒	☐	Clinical data
☒	☐	Dual use research of concern
☒	☐	Plants

Methods

n/a	Involved in the study	
☒	☐	ChIP-seq
☐	☒	Flow cytometry
☒	☐	MRI-based neuroimaging

Antibodies

Antibodies used

The following Antibody information is described as"Antibody (species/isotype), Source, Catalog number, Lot number, Dilution".
Details of antibodies are also available in supplementary tables 1 and 2.
GATA6 (D61E4) XP® Rabbit mAb (PE Conjugate) Cell Signaling Technology 26452 4 1:600
Rabbit (DA1E) mAb IgG XP® Isotype Control (PE Conjugate) Cell Signaling Technology 5742 17 1:600
Goat Anti-Human SNAIL R&D Systems AF-3639 XRS0217071 1:400
Goat IgG Isotype Control Thermo Fisher Scientific 31245 0960621 1:400
APC anti-human CD324 (E-Cadherin) Antibody Biolegend 324108 B379257 1:100
APC Mouse IgG1, κ Isotype Ctrl (FC) Antibody Biolegend 400122 B379803 1:100
Recombinant Anti-Transcription factor AP-2-alpha antibody [EPR2688(2)] Abcam ab108311 1014077-1 1:200
Recombinant Rabbit IgG, monoclonal [EPR25A] - Isotype Control Abcam ab172730 1011223-6 1:200
Alexa Fluor 555 donkey anti-rabbit IgG Thermo Fisher Scientific A-31572 1866859 1:1000
Alexa Fluor 488 donkey anti-goat IgG Thermo Fisher Scientific A-11055 1827671 1:1000
NANOG (goat IgG) R&D Systems AF1997 KKJ0922031 1:400
OCT4 (mouse IgG) Santa Cruz sc-5279 C3121 1:600
BLIMP1 (rabbit IgG) Cell Signaling Technology 9115S 6 1:100
MEIS2 (mouse IgM) Novus Biologicals H00004212-M01 M3011-EH4 1:200
E-Cadherin (mouse IgG)   Abcam ab76055 GR3360021-7 1:200
BST2 (rabbit IgG) Abcam ab243230 1022489-8 1:200
Active β-Catenin (rabbit IgG) Cell Signaling Technology 19807S 4 1:400
Phospho-Smad1/Smad5/Smad9 (rabbit IgG) Cell Signaling Technology 13820S 3 1:400
GATA6 (goat IgG) R&D Systems AF1700 KWT0523031 1:600
GATA6 (rabbit IgG) Cell Signaling Technology 5851S 5 1:600
SOX17 (goat IgG) R&D Systems AF1924 KGA08150422 1:600
SOX17 (rabbit IgG) Cell Signaling Technology 81778S 1 1:500
CK7/KRT7 (rabbit IgG) Abcam ab181598 GR3321316-17 1:500
NR2F2 (rabbit IgG) Abcam ab211776 1006695-11 1:200
TFAP2C (mouse IgG) Santa Cruz sc-12762 D2717 1:400
Brachyury/T (goat IgG) R&D Systems AF2085 KQP0719121 1:400
Brachyury/T (rabbit IgG) Cell Signaling Technology 81694S 1 1:800
HAVCR1 (goat IgG) R&D Systems AF1750 GTB0618111 1:200
TFAP2A (mouse IgG) Santa Cruz sc-12726 L2619 1:400

SNAIL (goat IgG) R&D Systems AF-3639 XRS0217071 1:400
VIMENTIN (mouse IgG) eBioscience 14-9897 2297463 1:2000
VIMENTIN (rabbit IgG) Abcam ab137321 GR3425580-7 1:1000
CD34 (rabbit IgG) Abcam ab81289 1071545-11 1:100
CD31 (mouse IgG) Abcam ab9498 1035700-12 1:100
LUM (rabbit IgG) Invitrogen MA5-29402 35FC7A02 1:100
DCN (rabbit IgG) Abcam ab151988 GR122269-56 1:100
FLT1 (rabbit IgG) Abcam ab32152 GR3321125-17 1:200
FOXA1 (mouse IgG) Abcam ab55178 GR3448710-4 1:100
KDR (goat IgG) R&D Systems AF357 CUE0620061 1:400
Alexa Fluor 488 donkey anti-rabbit IgG Jackson Immunoresearch 711-545-152 139808 1:600
Alexa Fluor 568 donkey anti-mouse IgG Thermo Fisher Scientific A-10037 1827879 1:600
Alexa Fluor 488 donkey anti-mouse IgG Thermo Fisher Scientific A-21202 1820538 1:600
Alexa Fluor 647 donkey anti-rabbit IgG Thermo Fisher Scientific A-31573 1874788 1:600
Alexa Fluor 555 donkey anti-rabbit IgG Thermo Fisher Scientific A-31572 1866859 1:600
Alexa Fluor 647 donkey anti-goat IgG Thermo Fisher Scientific A-21447 1841382 1:600
Alexa Fluor 488 donkey anti-goat IgG Thermo Fisher Scientific A-11055 1827671 1:600

Validation

All the antibodies have been validated by the companies from which they were offered. This information was used for further validate of the antibodies used in this work. Details of the validation statements, antibody profiles and relevant citations can be found on the manufacturer's website.
All the antibodies used in this work are for immunostaining purpose only. We list the immunostaining validation of the manufactures and the number of citations as follows:
GATA6 (D61E4) XP® Rabbit mAb (PE Conjugate): https://www.cellsignal.cn/products/antibody-conjugates/gata-6-d61e4-xp-rabbit-mab-pe-conjugate/26452. The antibody has been referenced in 7publications.
Rabbit (DA1E) mAb IgG XP® Isotype Control (PE Conjugate) : https://www.cellsignal.cn/products/antibody-conjugates/rabbit-da1e-mab-igg-xp-isotype-control-pe-conjugate/5742. The antibody has been referenced in 34 publications.
Goat Anti-Human SNAIL: https://www.rndsystems.com/cn/products/human-snail-antibody_af3639. The antibody has been referenced in 35 publications.
Goat IgG Isotype Control: https://www.thermofisher.cn/cn/zh/antibody/product/Goat-IgG-Polyclonal/31245. The antibody has been referenced in 13 publications.
APC anti-human CD324 (E-Cadherin) Antibody: https://www.biolegend.com/en-gb/products/apc-anti-human-cd324-e-cadherin-antibody-3752. The antibody has been referenced in 18 publications.
APC Mouse IgG1, κ Isotype Ctrl (FC) Antibody: https://www.biolegend.com/en-gb/products/apc-mouse-igg1-kappa-isotype-ctrl-fc-3034. The antibody has been referenced in 57 publications.
Recombinant Anti-Transcription factor AP-2-alpha antibody [EPR2688(2)]: https://www.abcam.cn/products/primary-antibodies/transcription-factor-ap-2-alpha-antibody-epr26882-ab108311.html. The antibody has been referenced in 27 publications.
Recombinant Rabbit IgG, monoclonal [EPR25A] - Isotype Control: https://www.abcam.cn/products/primary-antibodies/rabbit-igg-monoclonal-epr25a-isotype-control-ab172730.html. The antibody has been referenced in 617 publications.
Alexa Fluor 555 donkey anti-rabbit IgG: https://www.thermofisher.cn/cn/zh/antibody/product/Donkey-anti-Rabbit-IgG-H-L-Highly-Cross-Adsorbed-Secondary-Antibody-Polyclonal/A-31572. The antibody has been referenced in 2378 publications.
Alexa Fluor 488 donkey anti-goat IgG : https://www.thermofisher.cn/cn/zh/antibody/product/Donkey-anti-Goat-IgG-H-L-Cross-Adsorbed-Secondary-Antibody-Polyclonal/A-11055. The antibody has been referenced in 3529 publications.
NANOG (goat IgG): https://www.rndsystems.com/cn/products/human-nanog-antibody_af1997. The antibody has been referenced in 311 publications.
OCT4 (mouse IgG): https://www.scbt.com/zh/p/oct-3-4-antibody-c-10. The antibody has been referenced in 2667 publications.
BLIMP1 (rabbit IgG): https://www.cellsignal.cn/products/primary-antibodies/blimp-1-prdi-bf1-c14a4-rabbit-mab/9115. The antibody has been referenced in 83 publications.
MEIS2 (mouse IgG): https://www.novusbio.com/products/meis2-antibody-1h4_h00004212-m01. The antibody has been referenced in 8 publications.
E-Cadherin (mouse IgG): https://www.abcam.cn/products/primary-antibodies/e-cadherin-antibody-m168-c-terminal-ab76055.html. The antibody has been referenced in 406 publications.
BST2 (rabbit IgG): https://www.abcam.cn/products/primary-antibodies/bst2tetherin-antibody-epr20202-150-ab243230.html. The antibody has been referenced in 2 publications.
Active β-Catenin (rabbit IgG): https://www.cellsignal.cn/products/primary-antibodies/non-phospho-active-b-catenin-ser45-d2u8y-xp-rabbit-mab/19807. The antibody has been referenced in 162 publications.
Phospho-Smad1/Smad5/Smad9 (rabbit IgG): https://www.cellsignal.cn/products/primary-antibodies/phospho-smad1-ser463-465-smad5-ser463-465-smad9-ser465-467-d5b10-rabbit-mab/13820. The antibody has been referenced in 517 publications.
GATA6 (goat IgG): https://www.rndsystems.com/cn/products/human-gata-6-antibody_af1700. The antibody has been referenced in 134 publications.
GATA6 (rabbit IgG): https://www.cellsignal.cn/products/primary-antibodies/gata-6-d61e4-xp-rabbit-mab/5851. The antibody has been referenced in 117 publications.
SOX17 (goat IgG): https://www.rndsystems.com/cn/products/human-sox17-antibody_af1924. The antibody has been referenced in 487 publications.
SOX17 (rabbit IgG): https://www.cellsignal.cn/products/primary-antibodies/sox17-d1t8m-rabbit-mab/81778. The antibody has been referenced in 25 publications.
CK7/KRT7 (rabbit IgG): https://www.abcam.cn/products/primary-antibodies/cytokeratin-7-antibody-epr17078-cytoskeleton-marker-ab181598.html. The antibody has been referenced in 116 publications.
NR2F2 (rabbit IgG): https://www.abcam.cn/products/primary-antibodies/nr2f2-antibody-epr18442-ab211776.html. The antibody has been referenced in 3 publications.
TFAP2C (mouse IgG): https://www.scbt.com/zh/p/ap-2gamma-antibody-6e4-4. The antibody has been referenced in 145 publications.
Brachyury/T (goat IgG): https://www.rndsystems.com/cn/products/human-mouse-brachyury-antibody_af2085. The antibody has been referenced in 263 publications.
Brachyury/T (rabbit IgG): https://www.cellsignal.cn/products/primary-antibodies/brachyury-d2z3j-rabbit-mab/81694. The antibody has been referenced in 39 publications.

HAVCR1 (goat IgG): https://www.rndsystems.com/cn/products/human-tim-1-kim-1-havcr-antibody_af1750. The antibody has been referenced in 37 publications.
TFAP2A (mouse IgG): https://www.scbt.com/zh/p/ap-2alpha-antibody-3b5. The antibody has been referenced in 97 publications.
SNAIL (goat IgG): https://www.rndsystems.com/cn/products/human-snail-antibody_af3639. The antibody has been referenced in 35 publications.
VIMENTIN (mouse IgG): https://www.thermofisher.cn/cn/zh/antibody/product/Vimentin-Antibody-clone-V9-Monoclonal/14-9897-80. The antibody has been referenced in 21 publications.
VIMENTIN (rabbit IgG): https://www.abcam.cn/products/primary-antibodies/vimentin-antibody-ab137321.html. The antibody has been referenced in 257 publications.
CD34 (rabbit IgG): https://www.abcam.cn/products/primary-antibodies/cd34-antibody-ep373y-ab81289.html. The antibody has been referenced in 570 publications.
CD31 (mouse IgG): https://www.abcam.cn/products/primary-antibodies/cd31-antibody-jc70a-ab9498.html. The antibody has been referenced in 263 publications.
LUM (rabbit IgG): https://www.thermofisher.cn/cn/zh/antibody/product/LUM-Antibody-clone-077-Recombinant-Monoclonal/MA5-29402. The antibody has been referenced in 4 publications.
DCN (rabbit IgG): https://www.abcam.cn/products/primary-antibodies/decorin-antibody-ab151988.html. The antibody has been referenced in 4 publications.
FLT1 (rabbit IgG): https://www.abcam.cn/products/primary-antibodies/vegf-receptor-1-antibody-y103-ab32152.html. The antibody has been referenced in 308 publications.
FOXA1 (mouse IgG): https://www.abcam.cn/products/primary-antibodies/foxa1-antibody-1b1-ab55178.html. The antibody has been referenced in 14 publications.
KDR (goat IgG): https://www.rndsystems.com/cn/products/human-vegfr2-kdr-flk-1-antibody_af357. The antibody has been referenced in 58 publications.
Alexa Fluor 488 donkey anti-rabbit IgG: https://www.jacksonimmuno.com/catalog/products/711-545-152. The antibody has been referenced in 1588 publications.
Alexa Fluor 568 donkey anti-mouse IgG: https://www.thermofisher.cn/cn/zh/antibody/product/Donkey-anti-Mouse-IgG-H-L-Highly-Cross-Adsorbed-Secondary-Antibody-Polyclonal/A10037. The antibody has been referenced in 1093 publications.
Alexa Fluor 488 donkey anti-mouse IgG: https://www.thermofisher.cn/cn/zh/antibody/product/Donkey-anti-Mouse-IgG-H-L-Highly-Cross-Adsorbed-Secondary-Antibody-Polyclonal/A-21202. The antibody has been referenced in 5504 publications.
Alexa Fluor 647 donkey anti-rabbit IgG: https://www.thermofisher.cn/cn/zh/antibody/product/Donkey-anti-Rabbit-IgG-H-L-Highly-Cross-Adsorbed-Secondary-Antibody-Polyclonal/A-31573. The antibody has been referenced in 2763 publications.
Alexa Fluor 555 donkey anti-rabbit IgG: https://www.thermofisher.cn/cn/zh/antibody/product/Donkey-anti-Rabbit-IgG-H-L-Highly-Cross-Adsorbed-Secondary-Antibody-Polyclonal/A-31572. The antibody has been referenced in 2378 publications.
Alexa Fluor 647 donkey anti-goat IgG: https://www.thermofisher.cn/cn/zh/antibody/product/Donkey-anti-Goat-IgG-H-L-Cross-Adsorbed-Secondary-Antibody-Polyclonal/A-21447. The antibody has been referenced in 1430 publications.
Alexa Fluor 488 donkey anti-goat IgG: https://www.thermofisher.cn/cn/zh/antibody/product/Donkey-anti-Goat-IgG-H-L-Cross-Adsorbed-Secondary-Antibody-Polyclonal/A-11055. The antibody has been referenced in 3529 publications.

Eukaryotic cell lines

Policy information about cell lines and Sex and Gender in Research

Cell line source(s)

Mouse embryonic fibroblast were acquired from Millipore (PMEF-CFL). DR4 MEFs were acquired from the Cell Bank of the Chinese Academy of Sciences (https://www.cellbank.org.cn). 293T (human embryonic kidney cells) were acquired from ATCC (CRL-3216). The three naive hESC lines (female AIC-N1, male AIC-N2, and male AIC-N4) and the three primed hESC lines (male hES1, male h1, and female H9) used in this study were established and obtained in our previous study (Ai, et al., Cell Research, 2023; Ai et al., Biomaterials, 2020). MESP1-mTomato knock-in reporter hESCs were provided by Dr. Jie Na (Tsinghua University).

Authentication

hESC lines were authenticated by genomic PCR, immunostaining, bulk RNA-seq, and in vitro differentiation tests. Moreover, Naive hESC lines (AIC-N1, AIC-N2, and AIC-N4) and the primed hESC lines (hES1, h1, and H9) were authenticated in previous study (Ai, et al., Cell Research, 2023; Ai et al., Biomaterials, 2020).

Mycoplasma contamination

All cell lines were routinely checked for mycoplasma contaminations using MycoAlert Mycoplasma Detection Kit (LONZA, LT07-318) every two weeks, and all cell lines used in this study have been ruled out of mycoplasma contamination.

Commonly misidentified lines
(See ICLAC register)

No commonly misidentified lines were used.

Plants

Seed stocks

Report on the source of all seed stocks or other plant material used. If applicable, state the seed stock centre and catalogue number. If plant specimens were collected from the field, describe the collection location, date and sampling procedures.

Novel plant genotypes

Describe the methods by which all novel plant genotypes were produced. This includes those generated by transgenic approaches, gene editing, chemical/radiation-based mutagenesis and hybridization. For transgenic lines, describe the transformation method, the number of independent lines analyzed and the generation upon which experiments were performed. For gene-edited lines, describe the editor used, the endogenous sequence targeted for editing, the targeting guide RNA sequence (if applicable) and how the editor was applied.

Authentication

Describe any authentication procedures for each seed stock used or novel genotype generated. Describe any experiments used to assess the effect of a mutation and, where applicable, how potential secondary effects (e.g. second site T-DNA insertions, mosiacism, off-target gene editing) were examined.

Flow Cytometry

Plots

Confirm that:

☒ The axis labels state the marker and fluorochrome used (e.g. CD4-FITC).

☒ The axis scales are clearly visible. Include numbers along axes only for bottom left plot of group (a 'group' is an analysis of identical markers).

☒ All plots are contour plots with outliers or pseudocolor plots.

☒ A numerical value for number of cells or percentage (with statistics) is provided.

Methodology

Sample preparation	To detect specific markers of ExM or AM lineages using flow cytometry, naive and primed hESC derivatives grown under different induced conditions were dissociated into single cells with 50% TrypLE, centrifuged, and washed with ice-cold DPBS containing 1% FBS (BI, 04-001-1A). Dissociated single cells were fixed with 4% paraformaldehyde at room temperature for 10 minutes, then washed three times with ice-cold DPBS containing 1% FBS and 100 mM glycine. Subsequently, permeabilization and blocking were performed with DPBS containing 0.2% Triton X-100 and 3% BSA at room temperature for 15 minutes. The live-cell staining for E-cadherin was performed without fixation and permeabilization. Cells were incubated at 4°C for 30 minutes with conjugated primary antibodies and their isotype control diluted in DPBS containing 1% BSA, and then washed three times with ice-cold DPBS containing 1% FBS. For unconjugated primary antibodies and their isotype control, cells were further stained at 4°C for 30 minutes with secondary antibodies diluted in DPBS containing 1% BSA, followed by three washes with ice-cold DPBS containing 1% FBS. For the sorting of MESP1-mTomato-positive primitive streak-like intermediates, wild-type CBAPs/CBANs differentiated for the same duration were used as negative controls.
Instrument	Flow cytometry was carried out using a FACSAria III.
Software	Data were analyzed using FlowJo software
Cell population abundance	For analyses, over 100,000 cells were gated each time for analyze.
Gating strategy	Preliminary FSC/SSC gating was selected by the scatter plots of normal cell population, and gate boundaries were determined by the positive/negative control and based on experience.

☒ Tick this box to confirm that a figure exemplifying the gating strategy is provided in the Supplementary Information.
